# Descripción clínica y genética de pacientes con enfermedad granulomatosa crónica en un hospital pediátrico

**DOI:** 10.7705/biomedica.7565

**Published:** 2024-12-23

**Authors:** Xareni Berriozábal-Villarruel, Guadalupe Fernanda Godínez-Zamora, Patricia Baeza-Capetillo, Uriel Pérez-Blanco, Sara Elva Espinosa-Padilla, Jesús Aguirre-Hernández, Lizbeth Blancas-Galicia, Omar Josué Saucedo-Ramírez

**Affiliations:** 1 Departamento de Alergología e Inmunología Clínica, Hospital Infantil de México Federico Gómez, Ciudad de México, México Hospital Infantil de México Federico Gómez Hospital Infantil de México Federico Gómez Ciudad de México México; 2 Laboratorio de Genómica, Genética y Bioinformática, Hospital Infantil de México, Federico Gómez, Ciudad de México, México Hospital Infantil de México Federico Gómez Hospital Infantil de México, Federico Gómez Ciudad de México México; 3 Laboratorio de Inmunodeficiencias, Instituto Nacional de Pediatría, Ciudad de México, México Instituto Nacional de Pediatría Instituto Nacional de Pediatría Ciudad de México México

**Keywords:** enfermedad granulomatosa crónica, NADPH oxidasa 2, estudios de seguimiento, vacuna BCG., Granulomatous disease, chronic, NADPH oxidase 2, follow-up studies, BCG vaccine.

## Abstract

**Introducción.:**

La enfermedad granulomatosa crónica es un error innato de la inmunidad caracterizado por aumento de la susceptibilidad a desarrollar infecciones por hongos y bacterias, e inflamación no regulada. Se produce por defectos en la coenzima NADPH oxidasa y la proteína EROS.

**Objetivo.:**

Caracterizar clínica y genéticamente cuatro pacientes con enfermedad granulomatosa crónica del Hospital Infantil de México Federico Gómez.

**Material y métodos.:**

Los pacientes con diagnóstico de enfermedad granulomatosa crónica diagnosticados por la técnica de oxidación de dihidrorrodamina, fueron caracterizados molecular y genéticamente mediante la medición de la expresión de las subunidades de la NADPH oxidasa, y por secuenciación y análisis de exorna. Se obtuvieron diferentes variables de la historia clínica y se describió cada una de ellas.

**Resultados.:**

Se describieron cuatro pacientes de sexo masculino con enfermedad granulomatosa crónica. En tres se encontró mutación del gen *CYBB*: dos tuvieron variantes patógenas sin sentido y el otro tuvo deleción de este gen y genes contiguos; el cuarto paciente mostró ausencia de expresión de la subunidad p47^*phox*^ . Las madres de los tres pacientes con mutación en *CYBB*, resultaron portadoras. Los tres casos con alteraciones en *CYBB* presentaron infecciones graves y recurrentes, además de infección por el bacilo de Calmette-Guérin como manifestación inicial. El paciente con ausencia de p47^*phox*^ presentó el cuadro clínico menos grave. Por el contrario, la deleción de *CYBB* y otros genes contiguos se asoció con un mal pronóstico. Ninguno de los pacientes recibió trasplante de células progenitoras hematopoyéticas.

**Conclusiones.:**

En este grupo de pacientes, la enfermedad granulomatosa crónica causada por variantes patógenas en *CYBB* fue la más frecuente. A las madres portadoras se les debe hacer seguimiento clínico por el riesgo de manifestaciones inflamatorias, autoinmunitarias e infecciosas. Una de las primeras manifestaciones en los afectados fue la infección por el bacilo de Calmette-Guérin y, en países como México, en donde se aplica esta vacuna, los casos con reacciones adversas deben ser tamizados para descartar enfermedad granulomatosa crónica.

## Abstract (en)

**Introduction.:**

Chronic granulomatous disease is a congenital immune disorder characterized by increased susceptibility to fungal and bacterial infections and dysregulated inflammation. It is caused by defects in the NADPH oxidase and EROS protein.

**Objective.:**

To characterize clinically and genetically four patients with chronic granulomatous disease at the *Hospital Infantil de México Federico Gómez.*

**Materials and methods.:**

Patients diagnosed with chronic granulomatous disease by the dihydrorhodamine oxidase technique were molecularly and genetically characterized by measuring NADPH oxidase subunit expression and exorne sequencing and analysis. The different clinical variables were obtained from clinical files, and each case was described.

**Results.:**

We described four male patients with chronic granulomatous disease: two with pathogenic variants in *CYBB*, one with *CYBB* and adjacent genes deleted, and one without p47^*phox*^ expression. Mothers of the three patients with mutated *CYBB* were carriers. All three cases with *CYBB* had severe and recurrent infections in addition to Calmette-Guérin bacillus infection as the initial manifestation. The autosomal recessive case of p47^*phox*^ deficiency had the mildest clinical presentation. Deleting *CYBB* and several contiguous genes was associated with a poor prognosis. None of the patients received hematopoietic stem cell transplantation.

**Conclusions.:**

Chronic granulomatous disease, secondary to pathogenic variants in CYBB was the most common in these Mexican patients. The carrier mothers should be followed clinically because of the potential risk of inflammatory, autoimmune, and infectious manifestations. One of the first manifestations was Calmette-Guérin bacillus infection, and in countries such as Mexico, where this vaccine is administered, cases with any type of adverse reaction should be evaluated to rule out chronic granulomatous disease.

La enfermedad granulomatosa crónica se debe a un error innato de la inmunidad que se produce por defectos en la función de alguna de las cinco subunidades del complejo enzimático nicotinamida adenina dinucleótido fosfato oxidasa (NADPH), o por defectos en la proteína esencial para las especies reactivas de oxígeno (EROS) ^1^.

Los genes asociados con la enfermedad granulomatosa crónica son *CYBB, CYBA, NCF1, NCF2, NCF4 y CYBC1*. La enfermedad debida a alteraciones en *CYBB* tiene un patrón de herencia ligado al cromosoma X, mientras que la enfermedad granulomatosa crónica por mutaciones en los otros cuatro genes tiene una herencia autosómica recesiva ^2^. La enfermedad granulomatosa crónica confiere una mayor susceptibilidad a infecciones por hongos y bacterias, así como a hiperinflamación ^3^.

El tratamiento habitual consiste en evitar que los pacientes se infecten, administrándoles profilaxis antimicrobiana y antifúngica. El tipo de microorganismos que infecta a estos pacientes varía entre las diferentes zonas geográficas, por ejemplo, en Latinoamérica, las infecciones por micobacterias son más frecuentes que en países desarrollados ^4^^-^^6^. El único tratamiento curativo es el trasplante de células progenitoras hematopoyéticas ^7^.

La incidencia y la prevalencia de esta enfermedad en México se desconoce. Muchos de los casos no son diagnosticados por falta de conocimiento entre los médicos de primer contacto. En México, solo una institución ha reportado casos de pacientes con enfermedad granulomatosa crónica ^8^. La última información reportada por el Hospital Infantil de México Federico Gómez fue en 1995, producto de una tesis de posgrado, en la que se revisaron 25 historias clínicas, y únicamente tres pacientes presentaron enfermedad granulomatosa crónica en un periodo de 25 años. ^9^ El Hospital Infantil de México Federico Gómez está ubicado en la Ciudad de México, es uno de los hospitales de especialidades pediátricas más completos y atiende pacientes que carecen de seguridad social.

En este trabajo, se presenta el cuadro clínico de cuatro pacientes con enfermedad granulomatosa crónica, no emparentados, diagnosticados y tratados en la institución en los últimos 10 años.

## Materiales y métodos

Se incluyeron cuatro pacientes con diagnóstico de enfermedad granulomatosa crónica tratados en el Hospital Infantil de México Federico Gómez y con historia clínica completa. El diagnóstico de los pacientes se realizó mediante la técnica de 1,2,3-dihidrorrodamina y nitroazul de tetrazolio, usando los estímulos forbol 12-miristato 13-acetato (PMA) y zimosán opsonizado.

Para los estudios moleculares y genéticos, se extrajo sangre periférica de pacientes y controles, después de la firma del consentimiento informado. La expresión de las subunidades de la NADPH oxidasa se obtuvo mediante tinción intracelular por citometría de flujo en el Laboratorio de Inmunodeficiencias del Instituto Nacional de Pediatría. El ADN genómico se aisló a partir de una muestra de sangre periférica o de células del epitelio bucal.

Las variantes patogénicas se detectaron por secuenciación de exorna completo, en el equipo NextSeq™ 500 (Ilumina^®^). El análisis bioinformático se llevó a cabo en el Laboratorio de Genómica, Genética y Bioinformática del Hospital Infantil de México Federico Gómez. La detección de las mutaciones en las madres portadoras de la enfermedad granulomatosa crónica ligada al cromosoma X, se realizó por las técnicas de 1,2,3-dihidrorrodamina, amplificación de sondas tras ligación múltiple y secuenciación por el método de Sanger en el Laboratorio de Inmunodeficiencias del Instituto Nacional de Pediatría. Se revisaron las historias clínicas de los pacientes para obtener la información.

### Consideraciones éticas

Los pacientes se incluyeron en el estudio con el consentimiento informado de los padres. Se analizaron muestras de sangre por lo que se considera que esta investigación implica un riesgo mínimo para el paciente. El presente estudio ha sido registrado y aprobado con el número 019/2011 por la Comisión de Investigación y el Comité de Ética; y autorizado por la Dirección de Investigación según las normas vigentes del Instituto Nacional de Pediatría de la Ciudad de México, México.

## Resultados

Se identificaron cuatro pacientes con diagnóstico confirmado de enfermedad granulomatosa crónica. Todos tenían completa historia clínica y sus características se describen a continuación.

### Paciente 1

Se trata de un paciente de sexo masculino de cinco años y nueve meses de edad originario de la Ciudad de México, con una media hermana por el lado materno.

Presentó antecedentes de linfadenitis por el bacilo de Calmette-Guérin (BCG) al mes de nacimiento, diarrea crónica desde los tres meses y neumonías recurrentes, de focos múltiples, a los 12, 19, 27 y 38 meses (cuadro 1). Se sospechó una alergia a la proteína de la leche de vaca, por lo que se eliminó de la dieta, pero no presentó mejoría clínica.

**Cuadro 1.** Características demográficas, clínicas y de laboratorio en los pacientes con enfermedad granulomatosa crónica

	Paciente 1	Paciente 2	Paciente 3	Paciente 4
Provincia de origen	Ciudad de México	Estado de México	Hidalgo	Veracruz
Edad de inicio de los síntomas	1 mes	2 meses	8 días	6 años
Edad al momento del diagnóstico de EGC	9 meses	11 meses	5 meses	8 años
Edad actual	5 años, 9 meses	9 años, 7 meses	Pérdida de seguimiento	10 años, 3 meses
Manifestaciones iniciales de EGC	Diarrea crónica	Linfadenitis por BCG	Linfadenitis por BCG	Osteomastoiditis
	Linfadenitis por BCG			Absceso subperióstico
Índice de oxidación de la dihidrorrodamina	0,9	1	1,3	13,4
Días de hospitalización	840	600	30	30

EGC: enfermedad granulomatosa crónica; BCG: bacilo de Calmette-Guérin

En el cuadro hemático, se encontró: hemoglobina de 10,1 g/dl (11,6 - 12,6 g/dl), 33 % de hematocrito (31 - 36 %), 12,4 x 10^3^ leucocitos/ml (6 - 17,5 x 10^3^/ mi), 5.600 x 10^3^ neutrófilos/ml (1.500 - 8.500 x 10^3^/ml), 6.800 x 10^3^ linfocitos/ml (4.000 - 13.500 x 10^3^/ml) e IgG sérica de 2.300 mg/dl (215 - 704 mg/dl).

**Figura 1.**
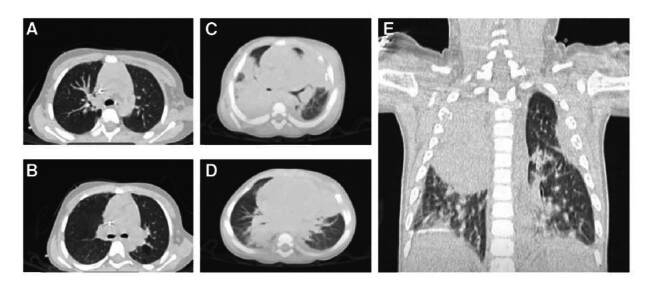
Tomografías de los pacientes con enfermedad granulomatosa crónica. A) Dilatación peribronquial (paciente 1). B) Engrasamiento de la cisura en el ápice derecho y zonas de neumonía con patrón de empedrado, de predominio inferior (paciente 1). C) Broncograma aéreo en los segmentos principales con áreas de consolidación bilateral en los lóbulos medio e inferior derechos (paciente 2). D) Engrasamiento interlobulillar con broncograma aéreo y tendencia a la consolidación, de predominio derecho (paciente 2). E) Imagen hiperdensa que abarca el lóbulo superior y medio, con engrasamiento pleural izquierdo y múltiples zonas de ocupación alveolar con patrón generalizado de vidrio esmerilado (paciente 2).

La tomografía computarizada de tórax reveló un absceso pulmonar apical derecho (figura 1A-B). Se tomó una biopsia de pulmón, a partir de la cual se aisló *Aspergillus fumigatus*, por lo que se sugirió aspergllosis invasiva. Además, según los criterios de Graham, se incluyó un diagnóstico probable de tuberculosis-mediante la prueba cutánea de derivado proteico purificado de 20 mm-, por lo cual se administró isonlacida durante nueve meses.

**Figura 2.**
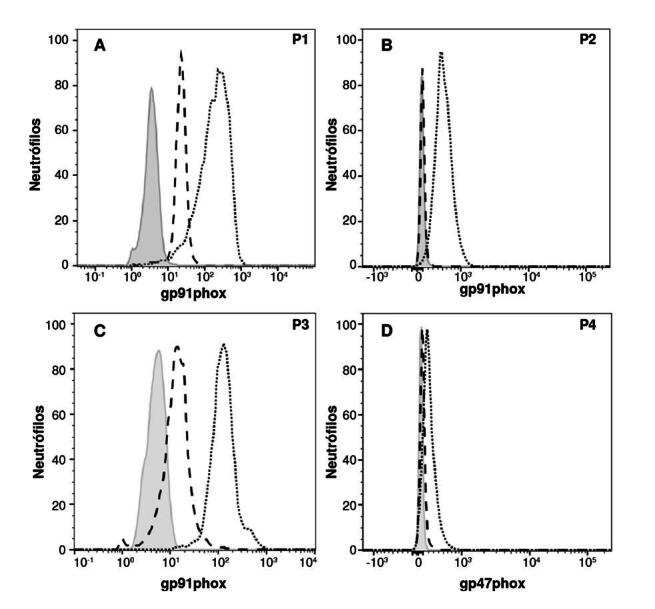
Expresión de las subunidades de la NADPH oxidasa por citometría de flujo en los pacientes con enfermedad granulomatosa crónica. El histograma gris sólido muestra la condición basal. Se muestran las subunidades de la NADPH oxidasa del paciente (línea discontinua) y del testigo (línea punteada). Se observa la expresión de gp91^*phox*^ en los pacientes 1 (A), 2 (B) y 3 (C), y la expresión de p47^*phox*^ , en el paciente 1.

Considerando los antecedentes infecciosos del paciente, se sospechó enfermedad granulomatosa crónica, la cual fue confirmada con las pruebas de nitroazul de tetrazolio y 1,2,3-dihldrorrodamina. Ambas mostraron ausencia de producción de especies reactivas de oxígeno y expresión nula de la subunidad gp91^*phox*^ (figura 2A).

**Figura 3.**
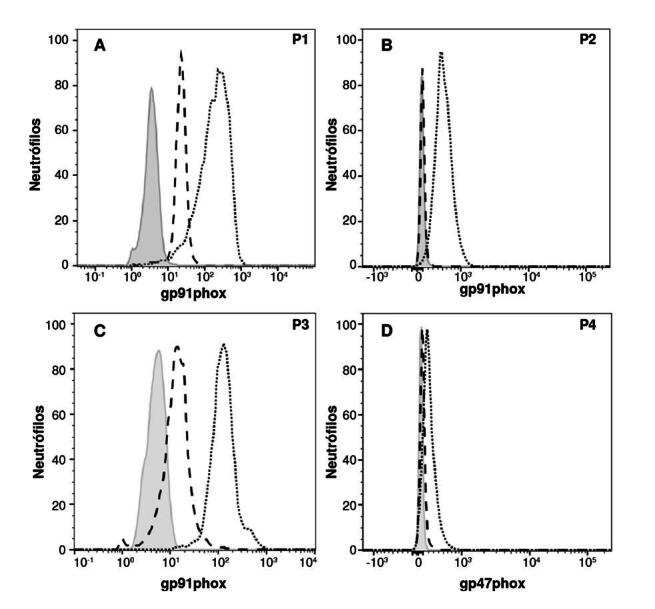
Árbol genealógico de los pacientes con enfermedad granulomatosa crónica: A) paciente 1, B) paciente 2 y C) paciente 3. Los números romanos Indican la generación; los cuadros negros representan al probando y, los círculos con punto, a los portadores de enfermedad granulomatosa crónica ligada al cromosoma X.

Se realizó secuenciación del exorna completo y se halló una variante patógena en el gen *CYBB*: c.676C>T (p.Arg226Ter), reportada previamente (10-24). En el estudio familiar, se encontró que su hermana tenía un fenotipo funcional (*Wild Type*, WT/WT) mientras que su madre resultó portadora de la misma variante (WT/c.676C>T), según la prueba de 1,2,3-dlhidrorrodamlna y la secuenciación por el método de Sanger (figura 3A).

Después del diagnóstico, se inició profilaxis con trimetroprim-sulfametoxazol, vorlconazol e Interferon gamma recomblnante (Imukin^®^). El paciente, que tenía peso y talla adecuados para su edad, había presentado otros eventos de neumonía, diarrea crónica (sugestivos de enfermedad inflamatoria de tipo Crohn) y osteomielitis en el astrágalo, ocasionada por cepas de *Klebsiella pneumoniae* productoras de (β-lactamasas de espectro extendido (BLEE+).

El paciente padece neumopatía crónica, requiere uso de oxígeno suplementario por las noches y no es candidato para recibir un trasplante de células progenitoras hematopoyéticas. Requirió en total 840 días de hospitalización.

### Paciente 2

Se trata de un paciente de sexo masculino de nueve años y siete meses de edad, originario del Estado de México, con el antecedente materno de tres familiares hombres fallecidos en la infancia.

El paciente recibió la vacuna de BCG cuando nació, dos meses después presentó una reacción adversa local y, a los cinco meses, una reacción diseminada en ganglios axilares y cervicales, motivo por el cual fue referido al Hospital Infantil de México Federico Gómez (cuadro 1).

Se practicó una biopsia de ganglios cervicales. El estudio microbiológico resultó en prueba Xpert^®^ MTB/RIF (GeneXpert^®^) positiva para el complejo Mycobacterium tuberculosis y en el cultivo en medio Lowenstein-Jensen creció M. bovis.

Los valores del cuadro hemático estuvieron dentro de los rangos normales para la edad: hemoglobina de 13,1 g/dl (11,6 -12,6 g/dl), 39,1 % de hematocrito (31 - 36 %), 8,1 x 10^3^ leucocitos/ml (6 -17,5 x 10^3^/ml), 3.160 x 10^3^ neutrófilos/ml (1.500 - 8.500 x 10^3^/ml), 4.050 x10^3^ linfocitos/ml (4.000 - 13.500 x 10^3^/ml), e IgG sérica de 1.800 mg/dl (215 - 704 mg/dl).

Durante la misma hospitalización, se practicó una tomografía computarizada pulmonar por deterioro respiratorio secundario a neumonía, en la cual se observaron imágenes de lesiones nodulares (figura 1C-E). Además, se detectó galactomanano en el suero y presencia de *A. fumigatus* en el líquido broncoalveolar, confirmándose una aspergilosis pulmonar invasiva.

Dadas las infecciones graves y recurrentes, se sospechó de un error innato de la inmunidad, específicamente, enfermedad granulomatosa crónica. En las pruebas de nitroazul de tetrazolio y 1,2,3-dihidrorrodamina no se detectó producción de especies reactivas de oxígeno, ni tampoco expresión de la subunidad gp91^*phox*^ (figura 2B). Mediante secuenciación de exorna completo, se halló una deleción de 30 genes contiguos, incluyendo *CYBB* y *XK*. Los resultados de las pruebas de 1,2,3-dihidrorrodamina y amplificación de sondas tras ligación múltiple, determinaron que la madre y la abuela materna eran portadoras de la deleción (figura 3B).

Se inició profilaxis con trimetroprim-sulfametoxazol, itraconazol e interferon gamma recombinante. Después del diagnóstico, el paciente presentó múltiples abscesos perianales (probable enfermedad inflamatoria de tipo Crohn) y neumonías recurrentes. También, se aisló *K. pneumoniae, K. oxytoca y Serratia marcescens*.

Actualmente, el paciente presenta fibrosis pulmonar, lo cual le impide ser candidato a un trasplante de células progenitoras hematopoyéticas. Requirió 600 días de hospitalización y tenía talla baja y peso adecuado para su edad.

### Paciente 3

Se trata de un paciente de sexo masculino de tres años y cinco meses de edad, originario del Estado de Hidalgo, con el antecedente de un hermano fallecido a los 11 meses de edad.

A los ocho meses de edad, el paciente presentó infección local y regional de ganglios cervicales, secundaria a la vacuna con el bacilo Calmette-Guérin, motivo por el que fue remitido al Hospital Infantil de México Federico Gómez (cuadro 1).

En el cuadro hemático, se encontró: hemoglobina de 12,1 g/dl (10,5 - 12 g/dl), 37 % de hematocrito (33 - 36 %), 10,2 x 10^3^ leucocitos/ml (6 - 17 x 10^3^/ mi), 4.150 (1.500-8.000 x 10^3^ neutrófilos/ml), 6.050 (4.000-13.500 x 10^3^ linfocitos/ml) e IgG sérica de 1.900 mg/dl (217 - 904 mg/dl).

Se resecó un ganglio cervical. El análisis de este tejido mediante la tinción de Ziehl-Neelsen, evidenció bacilos ácido-alcohol resistentes. La prueba Xpert^®^ MTB/RIF (Gene Xpert^®^) para el complejo *M. tuberculosis* resultó positiva.

Se le practicó una tomografía computacional de tórax que mostro una caverna apical, y áreas hiperdensas e irregulares en los segmentos pulmonares apical anterior derecho, Ungular inferior izquierdo y posterobasal izquierdo. Por lo anterior, se consideró una tuberculosis pulmonar diseminada y se inició tratamiento antituberculoso con pirazinamida, isoniacida, rifampicina y etambutol.

Por los antecedentes de un hermano fallecido en la infancia y la infección micobacteriana, se sospechó de enfermedad granulomatosa crónica. Este diagnóstico se confirmó mediante las pruebas de nitroazul de tetrazolio y 1,2,3-dihidrorrodamina, que también revelaron expresión nula de la subunidad gp91^*phox*^ (figura 2C). La secuenciación del exorna completo evidenció una variante patógena en *CYBB*: c.469C>T (p.Arg157Ter), reportada previamente ^25^^-^^32^. La prueba de 1,2,3-dihidrorrodamina y la secuenciación por el método de Sanger, mostraron que la madre era portadora de la mutación (WT/c.469C>T) (figura 3C).

Después del diagnóstico, el paciente presentó neumonía por Staphylococcus aureus y fue dado de alta con tratamiento profiláctico con trimetroprim-sulfametoxazol y voriconazol; sin embargo, el paciente no regresó a consulta.

### Paciente 4

Se trata de un paciente de sexo masculino de 10 años y tres meses de edad, originario de una comunidad endogámica del estado de Veracruz (cuadro 1).

A los seis años presentó otomastoiditis que requirió drenaje quirúrgico de un absceso subperióstico y colocación de tubos de drenaje. Refirió que esta lesión fue secundaria a un traumatismo en el pabellón auricular. No se aislaron microorganismos y fue remitido al Hospital Infantil de México Federico Gómez para descartar un error innato de la inmunidad.

En el cuadro hemático inicial se encontró: hemoglobina de 12,9 g/dl (11,5 -13,5 g/dl), hematocrito de 39 % (35 - 40 %), 8,8 x 10^3^ leucocitos/ml (4,5 - 13,5 x 10^3^/ml), 2.820 x 10^3^ neutrófilos/ml (1.500 - 8.000 x 10^3^/ml) y 4.580 x 10^3^ linfocitos/ml (1.500 - 6.800 x 10^3^/ml).

Se sospechó una enfermedad granulomatosa crónica, la cual se confirmó mediante las pruebas de nitroazul de tetrazolio y 1,2,3-dihidrorrodamina.

Al analizar las subunidades de la NADPH oxidasa, p47^*phox*^ resultó ausente (figura 2D). La secuenciación del exorna completo mostró ausencia del exón 2 de *NCF1*, gen que codifica para p47^*phox*^ . El paciente requirió 30 días de hospitalización, y tenía talla y peso bajos para su edad.

## Discusión

Los pacientes con enfermedad granulomatosa crónica son susceptibles de infecciones por micobacterias como *M. bovis* y *M. tuberculosis* en regiones geográficas específicas como México ^4^^,^^5^. En múltiples cohortes de pacientes con dicha enfermedad, a los que se les ha administrado la vacuna del bacilo de Calmette-Guérin, se han reportado complicaciones como BCG-osis y BCG-itis hasta en el 62 % de los casos ^5^.

La BCG-itis es más común, la linfadenitis y los abscesos en el sitio de vacunación son las manifestaciones más reportadas ^6^; la BCG-osis es una complicación sistémica y puede llegar a ser mortal. Los sitios más comunes de diseminación son los pulmones, el abdomen y los ganglios linfáticos ^8^. La reacción adversa a la vacuna de BCG se ha reportado como la primera manifestación clínica de la enfermedad granulomatosa crónica hasta en el 74 % de los casos ^8^. Todos los pacientes de este reporte tenían antecedentes de vacunación con BCG y tres presentaron infección secundaria al bacilo de Calmette-Guérin, local o sistémica.

Los pacientes con enfermedad granulomatosa crónica ligada al cromosoma X presentan trastornos inflamatorios que pueden ser, incluso, la primera manifestación de la enfermedad ^33^. El gastrointestinal es el sistema más afectado y sus manifestaciones clínicas son diarrea crónica, abscesos perianales, dolor abdominal, pérdida de peso, sangrado rectal, obstrucción intestinal y enfermedad inflamatoria intestinal ^3^. Los síntomas gastrointestinales pueden tener un amplio espectro; cuando son la manifestación inicial y no se acompañan de infecciones, difícilmente se asocian con enfermedad granulomatosa crónica ^3^. De los pacientes reportados aquí, uno desarrolló enfermedad inflamatoria intestinal con diarrea crónica, pero solo hasta que presentó infecciones se sospechó de enfermedad granulomatosa crónica.

La identificación molecular de las variantes patógenas es importante porque algunas se asocian con mayor gravedad de la enfermedad ^33^. La variante del paciente 1 (c.676C>T) originó un codón de parada prematuro en el aminoácido 226, por lo que las células no tienen una proteína funcional.

El paciente 2 presentó deleción del gen *CYBB* y otros genes contiguos (síndrome de McLeod). La secuenciación del exorna abarca únicamente las regiones codificantes y, por ello, los puntos de ruptura no pudieron establecerse con precisión. Sin embargo, se determinó que el punto de ruptura en el extremo 5’ está entre los exones 35 y 36 del gen *CFAP47* (transcrito ENST00000378653.8), y el del extremo 3’ está entre los exones 16 y 17 del gen SYTL5 (transcrito ENST00000297875.7). La deleción abarca entre 1.734.736 y 1.821.620 pares de bases, aproximadamente. El paciente 2 aún no ha presentado otras manifestaciones diferentes a las de la enfermedad granulomatosa crónica, asociadas con la deleción de los otros genes. Con la tecnología de secuenciación de nueva generación es posible detectar el síndrome de McLeod, tal como ocurrió en este caso.

La variante del caso 3 (c.469C>T) dio lugar a un codón de parada prematuro en el aminoácido 157, por lo que las células no tienen una proteína funcional. En el paciente 4 no fue posible determinar la variante patógena mediante la secuenciación del exorna, pues en este caso, *NCF1* es un pseudogén con una secuencia casi idéntica a la del gen funcional. Se requieren técnicas adicionales para identificar la mutación.

En el presente estudio, el análisis genético se extendió a las madres de los pacientes 1, 2 y 3, y se confirmó que son mujeres portadoras. Ellas deben ser vigiladas médicamente ya que tienen riesgo de padecer enfermedades autoinmunitarias, inflamatorias e, incluso, enfermedad granulomatosa crónica ^33^.

La detección de la variante patógena en el gen responsable en el paciente y su familia tiene implicaciones en el asesoramiento genético, y en el pronóstico y el tratamiento de la enfermedad ^8^. En los pacientes con deleción de genes contiguos, se debe medir el antígeno Kell, ya que el síndrome de McLeod está asociado con una deficiencia de las proteínas del sistema Kell. En caso de ausencia del antígeno, el individuo debe recibir un trasplante con el paquete globular Kell negativo. Por otro lado, el trasplante de células progenitoras hematopoyéticas en el síndrome de McLeod es controversial, aunque se han descrito casos exitosos ^34^.

Mientras un paciente no reciba un trasplante, se le debe administrar profilaxis antimicrobiana con trimetroprim-sulfametoxazol e itraconazol ^3^. El estafilococo es el agente etiológico mayoritariamente responsable de las infecciones y la aspergilosis invasiva es la complicación más letal en la enfermedad granulomatosa crónica ^8^.

En cada consulta, la vigilancia de la adhesión al tratamiento debe ser minuciosa, ya que se ha observado que solo es la adecuada cuando el paciente ya ha presentado uno o más eventos infecciosos graves que han requerido hospitalización. El interferón gamma recombinante se recomienda como profilaxis para las infecciones ^3^. Los dos pacientes que lo recibieron continuaron con infecciones o inflamación, pero los autores no tienen evidencia para concluir si fue útil o no su administración.

El trasplante de células hematopoyéticas se considera, hasta hoy, el único tratamiento curativo ^6^. Los pacientes incluidos en el presente estudio han sobrevivido sin este trasplante; sin embargo, han desarrollado secuelas en diferentes órganos, tienen baja calidad de vida y los costos generados por sus múltiples hospitalizaciones (mediana de 315 días por paciente) rebasan el costo del procedimiento de trasplante ^8^. Se sugiere la implementación del trasplante de progenitores hematopoyéticos en los pacientes afectados por enfermedad crónica granulomatosa en todos los hospitales de especialidades pediátricas.

## Conclusiones

La enfermedad granulomatosa crónica debe sospecharse en diferentes circunstancias clínicas-infecciosas e inflamatorias-, especialmente en pacientes masculinos, con antecedentes de familiares fallecidos en la infancia o sin ellos, reacción adversa a la vacuna del bacilo de Calmette-Guérin, linfadenopatías o neumonía.

El trasplante de células progenitoras hematopoyéticas debe considerarse en todo paciente con diagnóstico de enfermedad crónica granulomatosa, ya que disminuye la mortalidad y los costos hospitalarios a largo plazo.
